# Towards a Functional Cure for Diabetes Using Stem Cell-Derived Beta Cells: Are We There Yet?

**DOI:** 10.3390/cells10010191

**Published:** 2021-01-19

**Authors:** Stephanie Bourgeois, Toshiaki Sawatani, Annelore Van Mulders, Nico De Leu, Yves Heremans, Harry Heimberg, Miriam Cnop, Willem Staels

**Affiliations:** 1 Beta Cell Neogenesis (BENE) Research Group, Vrije Universiteit Brussel (VUB), 1090 Brussels, Belgium; Stephanie.An.B.Bourgeois@vub.be (S.B.); Annelore.An.Van.Mulders@vub.be (A.V.M.); Nico.De.Leu@vub.be (N.D.L.); Yves.Heremans@vub.be (Y.H.); Harry.Heimberg@vub.be (H.H.); 2 ULB Center for Diabetes Research, Medical Faculty, Université Libre de Bruxelles, 1070 Brussels, Belgium; toshiaki.sawatani@ulb.be (T.S.); mcnop@ulb.ac.be (M.C.); 3 Department of Endocrinology, University Hospital Brussels, 1090 Brussels, Belgium; 4 Department of Endocrinology, ASZ Aalst, 9300 Aalst, Belgium; 5 Division of Endocrinology, Erasmus Hospital, Université Libre de Bruxelles, 1070 Brussels, Belgium; 6 Service of Pediatric Endocrinology, Department of Pediatrics, KidZ Health Castle, Universitair Ziekenhuis Brussel (UZ Brussel), 1090 Brussels, Belgium

**Keywords:** beta cells, cell therapy, stem cells

## Abstract

Diabetes mellitus is a pandemic metabolic disorder that results from either the autoimmune destruction or the dysfunction of insulin-producing pancreatic beta cells. A promising cure is beta cell replacement through the transplantation of islets of Langerhans. However, donor shortage hinders the widespread implementation of this therapy. Human pluripotent stem cells, including embryonic stem cells and induced pluripotent stem cells, represent an attractive alternative beta cell source for transplantation. Although major advances over the past two decades have led to the generation of stem cell-derived beta-like cells that share many features with genuine beta cells, producing fully mature beta cells remains challenging. Here, we review the current status of beta cell differentiation protocols and highlight specific challenges that are associated with producing mature beta cells. We address the challenges and opportunities that are offered by monogenic forms of diabetes. Finally, we discuss the remaining hurdles for clinical application of stem cell-derived beta cells and the status of ongoing clinical trials.

## 1. Introduction

Pancreatic beta cells are critical regulators of blood glucose homeostasis by their unique ability to produce and secrete insulin in response to changing blood glucose levels. Because insulin is the only hormone that is able to decrease blood glucose levels, its release into the bloodstream must be strictly controlled in order to prevent blood glucose levels from surpassing dangerously low or high levels. The loss of beta cell function results in diabetes mellitus, a group of metabolic disorders that are characterized by chronically elevated blood glucose levels. Diabetes mellitus has reached epidemic proportions globally and it currently affects over 463-million people [1]. Although its two predominant forms are type 1 (T1D) and type 2 diabetes (T2D), rare forms of diabetes, including monogenic types that require specific attention, are increasingly diagnosed. T1D is marked by absolute insulin deficiency following autoimmune-mediated beta cell loss [2], while T2D is caused by relative insulin deficiency due to beta cell dysfunction, often in the context of peripheral insulin resistance [3]. Monogenic forms of diabetes result from single gene mutations and they are characterized by beta cell dysfunction, to varying degrees of severity [4]. Because no real cure exists for diabetes, daily insulin injections remain the standard of care for patients with T1D, late-stage T2D, and for a subset of patients with monogenic diabetes. Although this treatment is lifesaving, it conveys a chronic and costly burden of care, a persisting risk for acute and chronic complications, and it still results in an overall decreased life expectancy.

Beta cell replacement holds the potential to truly cure T1D and also possibly T2D and monogenic diabetes. Such cell therapy—through percutaneous infusion of pancreatic islets into the portal vein—is currently applied in some patients with brittle T1D [5], providing prolonged insulin independence. In selected patients, beta cell replacement proves to be superior to insulin administration with regard to overall metabolic control, prevention of severe hypoglycemia, and delaying the progression of micro- and macrovascular complications [6,7,8,9]. Despite this proof-of-principle for beta cell replacement as a genuine cure, donor islet transplantation is unattainable for the vast majority of diabetic patients for several reasons. First, donor islets are in short supply, which contrasts with the global disease prevalence. Donor shortage is further aggravated by a loss of up to half of grafted cells in the first few days after transplantation (reviewed in [10]). Delayed graft revascularization is one of the leading causes for this loss, since, following transplantation, islets enter a poorly vascularized and hypoxic microenvironment [11,12] that compromises islet cell and, in particular, beta cell survival and function. Excessive numbers of beta cells must be grafted to compensate for this early post-transplant cell loss, which further aggravates donor scarcity. Second, the allogenic origin of donor islet grafts necessitates lifelong immunosuppression, thereby increasing the susceptibility to infections and tumorigenesis (reviewed in [13]).

This donor islet shortage has fueled the search for alternative beta cell sources. Recent advances in differentiation protocols have positioned human embryonic stem cells (ESCs) and human induced pluripotent stem cells (iPSCs) as a promising and theoretically unlimited beta cell source. Currently, encapsulated pancreatic endoderm cells, differentiated from human ESCs, have already entered the first clinical trials (NCT03162926 (completed), NCT03163511 (recruiting), NCT02239354 (active, not recruiting), and NCT02939118 (enrolling by invitation)) and some preliminary results have been disseminated [14]. In this review, we define some key characteristics of adult beta cells and call attention to heterogeneity within the beta cell population. Next, we discuss the use of stem cells as an alternative beta cell source for transplantation. We elaborate on the current status of stem cell differentiation protocols in order to generate beta cells, the cues that are needed for functional beta cell maturation, and a number of hurdles that still need to be overcome. We address the following hurdles in detail: (i) the limited functional maturation of in vitro generated beta cells, (ii) the difficulties of graft survival upon transplantation due to the immune response and delay in graft revascularization, and (iii) safety concerns, especially regarding residual immature cells that may give rise to teratoma formation. Finally, we highlight the potential of gene-editing to generate patient- or disease-tailored beta(-like) cells for personalized medicine, discuss ongoing clinical trials, and offer some perspectives to the field.

## 2. Beta Cell Maturation and Heterogeneity

### 2.1. The Mature Beta Cell

Because the ultimate goal of stem cell differentiation protocols in diabetes research is to generate mature, fully functional beta cells, it is important to define what constitutes a functionally mature beta cell. The foremost fundamental characteristic of a beta cell lies in its ability to secrete appropriate amounts of insulin in response to glucose. Beta cells have a specialized machinery for fulfilling this role. In brief, glucose uptake by beta cells is mediated by specialized low affinity glucose transporters (mainly GLUT1 in human). Upon entry, glucose is phosphorylated by glucokinase (GCK) and is used to generate ATP through oxidative phosphorylation. This rise in ATP increases the ATP/ADP ratio, which, in turn, leads to the closure of ATP-sensitive potassium (K_ATP_) channels and cell membrane depolarization. The latter electric signal then triggers the opening of voltage-dependent calcium channels (VDCCs), leading to Ca^2+^ influx, resulting in the release of insulin granules through exocytosis [15,16]. Subsequently, beta cells generate a second, even larger, wave of insulin release (known as biphasic glucose-stimulated insulin secretion) that is K_ATP_-channel independent, but rather beta cell metabolism-dependent [17,18,19]. Mature beta cells can generate such responses—in a uniquely sensitive way—to minor physiological variations in plasma glucose levels.

Testing the maturity of beta cells with functional assays that interrogate the specialized cellular machinery that is implicated in glucose sensing and insulin secretion while using physiologically relevant variations in glucose concentrations is cumbersome, time-consuming, and often difficult. Therefore, cell markers have been used as surrogate measures for beta cell maturation. Typical beta cell maturation markers include the transcription factors GLIS3 [20], MAFA [21], NEUROD1 [22], NKX6.1 [23], PAX6 [24], PDX1 [25], SIX2 [26], and UCN3 [27,28]. Notably, besides positive regulators, beta cell maturity is equally determined by the absence of ‘disallowed’ or ‘forbidden’ genes that interfere with beta cell function including *Ldha*, *Mct1*, *SLC16A1*, *Hk1*, *Hk2*, and *Rest* (reviewed in [29]). The disallowed genes are upregulated under mild hyperglycemia as deduced from scRNA-seq data of beta cells isolated from diabetic rats, pointing to beta cell dysfunction occurring already in the early stages of diabetes [30]. In addition, one of the most important aspects of adult beta cells is their post-mitotic nature. Beta cell mass expansion mainly occurs shortly after birth by a process that rapidly declines with age [31,32], to reach an extremely low proliferation rate of 0.1–0.4% in the adult human [33,34]. Mathematical modeling of the accumulation of lipofuscin bodies in human beta cells suggests that, after the age of 20 years, long-lived beta cells age with the body [35,36].

### 2.2. Heterogeneity in the Adult Beta Cell Population

A next level of complexity with regard to the adult beta cell population is its heterogenous nature. The concept of beta cell heterogeneity was developed in the 1990s following metabolism-centered studies of dissociated cell populations, wherein some beta cells showed differential glucose-responsiveness [37,38] based on differences in glucokinase activity [39]. Recent studies unveiled particular underlying molecular hallmarks to provide compelling examples of beta cell heterogeneity. For example, Johnston et al. identified two distinct beta cell populations, ‘hub’ or ‘leader’ cells (<10% of beta cells), with pacemaker properties that orchestrate the electrophysiological responses of ‘follower’ cells (>90% of beta cells) upon insulin secretion challenges. Leader cells display high-potential mitochondria, high glucokinase expression, reduced insulin content, and lower expression of the beta cell markers PDX1 and NKX6.1 [40]. Bader et al. identified two distinct beta cell subpopulations that are based on the expression of Flattop (Fltp), a Wnt/planar cell polarity (PCP) effector, and reporter gene. Fltp^-^ beta cells are more proliferative and able to expand under conditions of increased metabolic demand, such as pregnancy, whereas Fltp^+^ beta cells are more mature, and they display higher insulin secretory capacity and increased mitochondrial function [41]. van der Meulen et al. identified immature beta cells in a neogenic niche at the adult islet periphery that lack UCN3 and are derived from transdifferentiated alpha cells [42]. Because these UCN3-negative beta cells only constitute~1.5% of the total beta cell population, they are likely distinct from the more abundant leader or follower cells and from the Fltp^+^ and Fltp^−^ beta cells, since the latter express UCN3 [41].

## 3. General Properties of Stem Cells

Stem cells are clonal cells with the potential to both self-renew and differentiate into a variety of functional somatic cells [43]. Stem cells are classified into three main types, according to their origin, potential, and developmental stage, as: (i) ESCs—which are isolated from the early mammalian embryo, (ii) adult stem cells—which can be found in various adult tissues (e.g., neural, hematopoietic, mesenchymal, and epidermal stem cells), and (iii) iPSCs—which are derived from adult cells that are reprogrammed back to an embryonic-like pluripotent state.

ESCs are highly undifferentiated cells that are isolated from the inner cell mass of mammalian blastocysts, i.e., early embryonic cells. ESCs can, on the one hand, proliferate indefinitely while maintaining their pluripotency and on the other differentiate into cells of all three germ layers [44]. In 1998, Thomson et al. established ESC lines from human blastocysts [45]. Because human ESCs can be maintained in culture for extended periods of time and can be differentiated into any desired target cell type, they represent a promising cell source for regenerative medicine to treat a host of diseases, including Parkinson’s disease, spinal cord injury, and diabetes [45]. However, even if functional cells and organs can be generated from ESCs, their transplantation remains subject to allograft rejection in the same manner as conventional donor organ transplants. Because human ESCs are derived from surplus human embryos, their application in regenerative medicine also raises ethical concerns that can be addressed by the use of iPSCs that are generated by reprogramming somatic cells, such as peripheral blood mononuclear cells or dermal fibroblasts, into the pluripotent state through the overexpression of a defined set of transcription factors (Oct4, Sox2, Klf4, and c-Myc) [46]. Such a straightforward method to produce human iPSCs directly from patients’ own cells opens the possibility of studying disease and screening drugs in vitro in a patient-specific manner. In addition, the inherent autologous nature of iPSCs provides them with unique immunological advantages over other cell sources in the context of cell therapy.

## 4. Current Status of Human ESC and iPSC Differentiation Protocols

The discovery of ESCs and iPSCs has opened the possibility to generate cells or tissues in vitro that can be used for the study of disease mechanisms, drug screening, and cell replacement. Regarding the latter, these cells promise an unlimited source of virtually any cell type that can be transplanted into patients, including pancreatic beta cells for people with diabetes.

### 4.1. Generation of Stem Cell-Derived Beta Cells

The main approach for differentiating stem cells into beta cells is by adherent cell culture with progressive, stepwise lineage commitment while using combinations of cues added to the culture medium. Using such an in vitro lineage differentiation approach, D’Amour et al. were the first to succeed in robust induction of definitive endoderm differentiation [47], with subsequent generation of pancreatic endocrine hormone-producing cells [48]. However, the endocrine cells that were generated were mainly polyhormonal (e.g., insulin and glucagon co-expressing cells) that are more akin to immature islet cells [49]. The resulting insulin-expressing cells also lacked the essential beta cell transcription factors NKX6.1 and PDX1 [50]. Over the ensuing years, strategies have been devised and optimized in order to generate monohormonal insulin-expressing cells that co-express NKX6.1 and PDX1 by modifying the composition and timing of growth factor and small molecule addition [51,52,53]. In 2014, Rezania et al. [53] and Pagliuca et al. [52] reported the successful generation of functional stem cell-derived beta-like cells that possessed many beta cell-specific traits, including glucose-responsive insulin secretion. Importantly, the transplantation of these cells was able to reverse diabetes in mice. However, the beta-like cells that were generated by these protocols [52,53] and follow-up studies [51,54,55,56] still displayed poor glucose-induced insulin secretion when compared to human islets.

More recently, Nair et al. [57] and Velazco-Cruz et al. [58] succeeded in generating functional human stem cell-derived beta cells showing dynamic glucose-stimulated insulin secretion that is similar to human islets. Nair et al. reaggregated immature human stem cell-derived beta-like cells into enriched-beta cell clusters after fluorescence-activated cell sorting (FACS) and demonstrated that the reaggregation/clustering is paramount to the generation of functionally superior beta cells with robust dynamic insulin secretion in vitro [57]. Mechanistically, the clustering of beta-like cells induced metabolic maturation by driving mitochondrial oxidative respiration, which is central to stimulus–secretion coupling in mature beta cells. This strategy not only increased the structural resemblance with native islets, but also the functional resemblance, both at the cellular (transcriptomic) and cluster (functional) level [57]. Velazco-Cruz et al. mainly focused on permitting TGF-β signaling during the final stage of differentiation (from endocrine progenitor to beta-like cell) and, additionally, on controlling cellular cluster size [58]. Although the TGF-β pathway is claimed to play a critical role during beta cell differentiation from stem cells [59], its role in beta cell function and insulin secretion remains controversial, as TGF-β signaling suppresses insulin transcription and reduces insulin protein levels and secretion [60], while others posited that TGF-β signaling is required for maintaining beta cell mass and regulating insulin secretion [61,62]. Further studies on TGF-β signaling in beta cells are needed, but, with respect to the differentiation of highly functional beta cells from stem cells, TGF-β fulfills a dual role, as its inhibition is required at early stages while its signaling is beneficial at late stages. Recently, Yoshihara et al. demonstrated that non-canonical WNT4 signaling drives the metabolic maturation of beta-like cells—essential for robust insulin secretion—in large part through the induction of an ERRgamma gene network [63], while Li et al. developed a beta cell differentiation protocol that is based on three previously published protocols [52,53,64] to obtain beta cells with high glucose-responsiveness and insulin production [65].

In addition to maturity, the purity of beta cells that are differentiated from human pluripotent stem cells must be taken into account for in vitro differentiation protocols, since a higher percentage of differentiated cells implies a lower percentage of contaminating immature and therefore possibly teratogenic cells. Sorting strategies while using a GFP reporter under the control of the insulin gene promoter or an antibody to the beta cell surface marker CD49a enabled obtaining 80–90% pure beta cells [57,66]. Alternatively, the cell-surface marker CD9 can be exploited for negative selection to enrich for glucose-responsive human beta-like cells [65]. These strategies may also prove to be useful for sorting other hormone-producing cells, such as alpha cells, which contribute to disease etiology by elevating blood glucose levels [67,68,69]. See Table 1 for a summary of the approaches for the generation and purification of stem cell-derived beta cells.

### 4.2. Generation of Non-Beta Pancreatic Endocrine Cell Types

Co-transplantation with other pancreatic endocrine cell types will likely benefit transplantation outcomes, as the release of insulin is regulated via complex paracrine interactions (discussed in more detail in the next chapter). To this end, efforts are ongoing for generating non-beta pancreatic endocrine cell types. In 2011, the directed differentiation of human ESCs into glucagon-positive cells, expressing the key alpha cell transcription factor ARX was reported [73]. Although these alpha-like cells secreted glucagon in vitro to some extent, proper regulation was lacking, which is indicative of their immature state [73]. More recently, Peterson et al. developed a differentiation protocol for the generation of alpha cells that express and secrete glucagon in response to low glucose and some glucagon secretagogues, and that elevate blood glucose levels upon transplantation in mice [74]. Efforts to develop differentiation protocols for somatostatin-producing delta cells, ghrelin-producing epsilon cells, and pancreatic polypeptide cells, and cell surface antibody-based sorting strategies will further contribute to the generation of islet-like clusters that will provide a unique resource for studying cell biology, disease modelling, drug screening, and cell replacement therapy.

## 5. Current Hurdles for Stem Cell Therapy and Possible Ways to Tackle Them

### 5.1. Functional Immaturity of Stem Cell-Derived Beta Cells

Beta cells reside within the pancreatic islets of Langerhans, being clustered together along with alpha, delta, epsilon, and pancreatic polypeptide cells. Beta cells intensively interact with a variety of cell types in their microenvironment, including the other endocrine and non-endocrine cells within the islet, but also with exocrine cells outside the islet. The numerous signals that beta cells receive are crucial for their functionality, survival, differentiation, and proliferation. First, beta-to-beta cell interactions are of major importance for a properly coordinated and synchronized insulin secretory response and for insulin gene expression, storage, biosynthesis, and release [75,76,77]. Paracrine cell interactions between different endocrine islet cell types are also paramount in fine-tuning the islet secretory response. [78,79]. Indeed, glucagon is a well-known regulator of insulin secretion, whereas somatostatin inhibits both insulin and glucagon secretion [27]. The importance of inter-endocrine cell-to-cell contacts for beta cell maturation is demonstrated by the enhanced maturation of human stem-cell-derived beta cells upon in vitro mimicking of cell clustering [57]. Therefore, the transplantation of islet-like clusters, rather than just beta cells alone, is likely to benefit glycemic control. Besides interactions with other endocrine cells, beta cells also receive signals from the extracellular matrix [80], endothelial cells [81], pericytes [82], neurons [83,84], and immune cells [85].

All of these cues from the islet microenvironment are essential for beta cell maturation. Notably, differentiation does not equal maturation, as Potten and Loeffler stated in 1990: “*Differentiation can be defined as a qualitative change in the cellular phenotype that is the consequence of the onset of synthesis of new gene products, that lead ultimately to functional competence. Maturation in contrast can be regarded as a quantitative change in the cellular phenotype or the cellular constituent proteins leading to functional competence*” [86]. This notion implies that, even when stem cell-derived beta cells express all known adult beta cell markers and produce high levels of insulin, they are not *per se* functionally mature, since the proof of functionality should be sought in functional tests that dynamically interrogate the beta cell glucose sensing and insulin secretory machinery.

It is very difficult, if not impossible, to provide all of the signals that beta cells receive from their microenvironment through simplified in vitro differentiation protocols, which is why current protocols rely on in vivo maturation for beta cells to become functional [52,53]. An alternative approach seeks not to generate mature cells, but uses early definitive endoderm or pancreatic progenitor cells and relies strongly on self-directed differentiation and maturation in order to obtain a functional stem cell-derived beta cell mass in vivo [70,87,88]. This is the strategy that was also chosen in the ongoing clinical trials, as mentioned in the introduction. However, while using this approach, it will take several months for the beta cells to become functional and this black box of in vivo maturation does not allow for an easy dissection of its mechanisms. Augsornworawat et al. performed single-cell RNA sequencing on human iPSC- and ESC- islet grafts that were transplanted for six months in diabetic mice and compared their gene expression profiles to stage 6 [89] ungrafted hPSC- islets and cadaveric human islets. The analyses confirmed that transplanted stem cell-derived beta cells possessed insulin secretory capacity and acquired expression of the beta cell maturation markers *INS, G6PC2, MAFA*, *MNX1, SIX2,* and *UCN3* [90]. Such studies provide a comprehensive resource for understanding human beta cell maturation and to improve differentiation strategies.

Ensuring quick islet revascularization following transplantation will likely benefit beta cell survival and functionality. Endothelial cell-derived signals are crucial for embryonic beta cell development [81,91] and for adult beta cell proliferation, survival, differentiation, and function [81]. Hypovascularization is not only one of the factors contributing to islet cell loss following transplantation, but it may also lead to beta cell dedifferentiation, since intraportal implants of human primary beta cells lose their mature phenotype and expression of key beta cell markers [92]. Strategies for improving graft revascularization have focused on the delivery of pro-angiogenic factors, primarily vascular endothelial growth factor A (VEGF-A), to islet cells. VEGF-A is a key angiogenic factor that is secreted by beta cells to promote endothelial cell migration, proliferation, and survival, and to regulate vascular permeability [81]. VEGF-A protein can be delivered in many ways, but one particularly interesting approach is by the transfection of *Vegfa* mRNA. Not only is this approach much safer when compared to viral-vector based gene delivery methods, its inherent short-term expression is beneficial as compared to long-term expression [93], which is detrimental for islet function and survival [94]. Recent work from our group showed that liposome-mediated transfection of human and mouse islet cells with synthetic modified *Vegfa* mRNA improves graft revascularization and increases beta cell mass [95].

Taken together, signals from the islet microenvironment are crucial in ensuring proper beta cell maturation and functionality. The translation of these insights to stem cell differentiation protocols is expected to improve transplantation outcomes.

### 5.2. Risk of Tumorigenesis

The therapeutic potential of pluripotent stem cells is vast, and it promises to transform medicine. The capacity of stem cells to self-renew and differentiate into any desired cell type underlies this promise, but these desirable features also bring along dangers, such as a risk of developing tumors. Therefore, several methods and tools have been developed for guaranteeing safety in therapeutic applications of human ESCs and iPSCs. First, tumorigenesis can be prevented by transplanting only differentiated cells. By using techniques, such as endocrine cell clustering and targeting specific signaling, recent differentiation protocols aim to generate higher percentages of mature functional beta cells and minimize the number of undifferentiated progenitor cells, as discussed above. Thus, protocol optimization in terms of improved differentiation minimizes the risk for tumorigenesis. Second, approaches for eliminating or sorting out undifferentiated human pluripotent stem cells in vitro have been developed, such as the use of chemical inhibitors [96], immunological targeting of undesired cell types [97,98,99], or the introduction of suicide-genes in the stem cell genome [100,101].

Chemical eradication of undifferentiated stem cells is possible by adding small molecules to the culture medium to selectively kill pluripotent cells. A high-throughput screen identified inhibitors of the key oleate biosynthesis enzyme stearoyl-CoA desaturase (SCD1) as agents that specifically compromise stem cell viability [96]. Knowing that SCD1 is abundantly expressed in iPSC-beta cells and required for beta cell identity, the effect of these SCD1 inhibitors on beta cell function warrants further investigation [102]. The immunological targeting of undifferentiated stem cells relies on the use of (cytotoxic) antibodies that are directed against specific stem cell markers to either kill them or separate them from differentiated cells. SSEA-5 glycan, for example, is a human ESC surface marker that has been used in order to selectively remove undifferentiated teratoma-forming cells prior to transplantation [97]. Gene editing can also aid in the removal of undifferentiated human pluripotent stem cells. In an innovative approach to selectively remove tumorigenic cells, two suicide gene cassettes were introduced in ESC-derived beta cells. The first safety cassette was a herpes simplex virus thymidine kinase (HSV-TK) that was driven by the telomerase gene promoter that is selectively active in undifferentiated cells, the second a nitroreductase (NTR) flanked by two loxP-sites, which is removed upon Cre-expression that is driven by the human insulin gene promoter. HSV-TK and NTR are sensitive to, respectively, ganciclovir and CB1954, which enables the elimination of tumorigenic undifferentiated cells at any desired moment, both in vitro and in vivo [100]. Finally, engineered human iPSCs with an inducible *Caspase-9* suicide gene have been developed, in which the small molecule chemical inducer of dimerization can effectively induce apoptosis in >99% of the cells [101]. While these methods are innovative and promising, they are not a validated approach for guaranteeing safety after clinical transplantation. See Table 2 for an overview of the possible approaches to prevent teratoma formation.

### 5.3. Graft Immune Rejection

Another major hurdle for the clinical application of stem cell-derived beta cells is immune rejection of the graft due to human leukocyte antigen (HLA)-mismatching. Patients currently depend on immune-suppressive drugs for preventing allograft immunity. These drugs can have side-effects, some minor, such as mouth ulcers, diarrhea, and acne, and some serious, including an increased risk for severe infection and malignancy (reviewed in [13]). Allograft rejection could be prevented if patient-specific iPSC derivates are transplanted, which guarantee perfect HLA-matching. However, the production of such personalized cells for individual patients would be very costly, time consuming, and is unlikely to become the universal solution for diabetes in the short term. To this end, HLA banks are currently being set up [103]. These banks contain a selected number of cell lines with homozygous HLA haplotypes that have been carefully chosen to match the majority of the population. The establishment of cell line banks decreases the number of cell lines required for matching to allow transplantation between a genetically unrelated donor and recipient and would thus make stem cell therapy more widely accessible [103,104,105].

Alternatively, regulatory T-cell therapy represents an attractive approach to establish immune tolerance to allogeneic grafts and to bypass ongoing autoimmunity upon transplantation. The administration of regulatory T-cells to new-onset diabetic mice can delay autoimmune diabetes and it prolongs islet allograft survival [106]. Clinical trials in T1D patients have already documented the therapeutic efficiency and safety of this approach [107,108]. Human regulatory T-cells can be isolated, cultured, and expanded ex vivo before transplantation; however, manufacturing the cells in therapeutically relevant numbers while maintaining high purity remains challenging (reviewed in [109,110]). Regulatory T-cells secrete multiple anti-inflammatory cytokines, some of which are in clinical use. Recently, beta cells have been engineered while using CRISPR/Cas9 to secrete the anti-inflammatory cytokine interleukin-10 (IL-10). The IL-10 gene was knocked into the C-peptide locus of the *Ins1* gene, which results in its transcription, translation, and secretion in a glucose-dependent manner [111]. IL-10, being delivered via an adeno-associated viral vector, is also known to promote islet graft survival upon transplantation in diabetic mice [112].

Another strategy for inducing immune tolerance involves the co-engraftment of selected cell types, such as mesenchymal stem cells (MSCs), which possess beneficial characteristics, including angiogenetic potential and the modulation of the immune response. Therefore, it is not surprising that the co-transplantation of islets with MSCs improves graft survival [113]. Studies that were performed in different experimental models, ranging from mouse [114,115], rat [116], and even non-human primate [117], illustrated that the co-engraftment of islets with MSCs prevents immune rejection through the inhibition of effector T-cells, expansion of regulatory T-cells and decreased pro-inflammatory cytokine production. In a clinical setting, islet transplantation is performed via the hepatic portal vein. However, this strategy does not allow for co-engraftment with MSCs, since the transplanted islets will lodge into the hepatic microcirculation, while the MSCs would end up in the lungs due to their small size. Therefore, the clinical applicability of this approach remains limited. The pre-treatment of islets with a cell-free cocktail of MSC-derived products has been demonstrated to suppress the immune response and promote transplantation outcomes (reviewed in [118]). This strategy can be easily implemented in transplantation protocols and it surpasses logistic and safety-related disadvantages of MSC co-engraftment [118,119,120].

**Figure 1:**
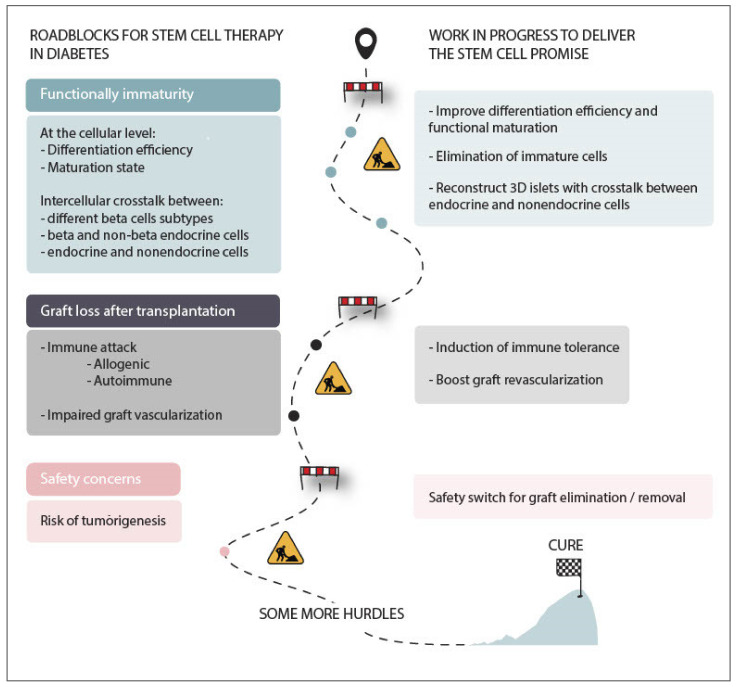
Roadblocks for a functional stem cell-based cure for diabetes. Stem cell-derived beta cells represent a promising alternative beta cell source for transplantation with the potential to cure various forms of diabetes. Nonetheless, major hurdles refrain stem cell-derived beta cells from providing a universal cure for diabetes. The functional maturation of stem cell-derived beta cells remains inferior compared to genuine beta cells, most likely due to the discrepancy of the simplified in vitro differentiation protocols with the complex, dynamic interactions that beta cells encounter in their natural microenvironment throughout development. Generating islet-like clusters containing different endocrine cell types, rather than clusters of only beta-like cells, will likely benefit the functionality of these clusters. Notably, following transplantation, a major donor cell loss occurs mainly due to insufficient graft revascularization and allo–and (auto)immunity. These hurdles can be tackled through delivery of pro-angiogenic factors that boost graft revascularization and by inducing immune tolerance to the graft and countering the underlying autoimmunity. Finally, teratoma formation remains a concern for stem cell therapy to become a therapeutic reality. Strategies to eliminate remaining undifferentiated stem cells need fine-tuning as well as methods to ensure easy graft retrieval in order to warrant the safety of stem cell-derived beta cell grafts.

Alternatively, interference with HLA gene expression can render human pluripotent stem cells hypoimmunogenic. By CRISPR/Cas9-mediated deletion of the accessory chain beta-2-microglobulin, cells lacking HLA class I molecules can be generated [121]. In addition, the inactivation of the *CIITA* gene, which encodes the ‘class II, major histocompatibility complex, transactivator’, can inhibit HLA class II expression [122]. Unfortunately, the deletion of beta-2-microglobulin also prevents HLA-E and HLA-G surface expression, which are essential in sustaining tolerance to natural killer cells. For this reason, Han et al. only deleted HLA-A/B- and C-genes in order to prevent CD8+ T cell-mediated cytotoxicity while maintaining natural killer cell tolerance [123]. Besides knocking out the HLA-A/-B/-C and *CIITA* genes while using multiplex genome editing, they additionally introduced the immunomodulatory factors PD-L1, HLA-G, and CD47. CD47 is a macrophage ‘do not eat me’ signal that prevents cell engulfment by macrophages [123]. A selection of attractive strategies to tackle immune rejection of stem cell-derived cellular grafts is compiled in Table 3. Additionally, see Figure 1 for a schematic summary of the current hurdles for stem cell therapy and their possible solutions.

### 5.4. The Emergence of Encapsulation Devices

The encapsulation devices have emerged as versatile methods to both enable the removal of transplanted human pluripotent stem cell-derived beta cells and physically shield grafted cells from immune attacks while still allowing for glucose sensing and insulin secretion. In encapsulation strategies, islet cells are embedded in a semipermeable gel-like structure that limits access to immune cells, but still allows for oxygen, nutrient, and hormone transport. Microencapsulation devices are made of biomaterials, such as alginate, while macroencapsulation devices are composed of polymers, such as polycaprolactone or polytetrafluoroethylene [130]. Both of the methods have their advantages and limitations. Microencapsulation devices allow for easier nutrient and oxygen exchange when compared to macroencapsulation devices due to their higher surface area to volume ratio. Whereas macroencapsulation devices are safer, as they are more readily retrievable, and, since a single device can house more cells, membrane parameters, such as pore size and porosity, can be better controlled. The downside here is that this comes at the expense of oxygen and nutrient exchange with individual cells, especially in the core of the graft [131].

## 6. Towards a Cure

### 6.1. The Paradigm of Gene-Editing for Monogenic Diabetes

Gene editing technologies have been developed for almost four decades, since Smithies et al., in 1985, used bacterial restriction endonucleases to create recombinant DNA [132]. Gene-editing endonucleases, such as zinc-finger nucleases, TAL effector nucleases, and CRISPR (clustered regularly interspersed short palindromic repeats)/Cas9 (CRISPR-associated protein 9), are revolutionizing genetic engineering in vitro and in vivo and they reach the efficiencies and precision required for clinical use. CRISPR/Cas9, in particular, is one of the hottest technologies and the favorite designer nuclease, because it does not involve producing target-specific proteins and only requires adjusting a short region of the single-guide RNA to achieve target specificity. This technology provides an innovative resource to generate various types of cells for disease modeling, drug screening, and new therapies in combination with stem cell technology.

Monogenic forms of diabetes are caused by mutations in genes that are involved in beta cell development, function, and survival [50,133]. Several groups have compared monogenic diabetes patients’ and healthy controls’ iPSC-derived beta cells. iPSCs from patients with an *HNF4A* mutation, causing autosomal dominant diabetes, differentiated similarly to healthy control iPSCs, and the iPSC-beta cells showed no obvious quantitative proteome differences [134]. Autosomal dominant *HNF1B* mutations result in diabetes and pancreatic hypoplasia [135]. Teo et al. showed that the expression of the pancreatic progenitor markers PDX1, FOXA2, and GATA4/6 is increased in *HNF1B* mutant cells, while PAX6 is decreased [136]. The heterozygous or homozygous *HNF1A* knockout in ESCs by CRISPR/Cas9 dose-dependently altered endocrine hormone expression, increased alpha cell gene expression, and impacted beta cell metabolism and insulin secretion [137]. McGrath et al. used CRISPR/Cas9 in ESCs to disrupt NEUROG3, which is associated with permanent neonatal diabetes mellitus [138,139,140,141], and showed that NEUROG3 is required for human pancreatic endocrine cell development [142]. Interestingly, Saarimäki-Vire et al. demonstrated that activating *STAT3* mutations, a cause of neonatal diabetes associated with beta cell autoimmunity [143], cause premature endocrine differentiation of patient’s iPSCs through the direct premature induction of *NEUROG3* expression. The differentiation phenotype was reversed when the *STAT3* mutation was corrected with CRISPR/Cas9 [144]. Activating mutations in *KCNJ11*, the pore-forming subunit of the K_ATP_ channel, also cause neonatal diabetes [145,146,147]. The biallelic *KCNJ11* mutation in human ESCs impairs insulin secretion that is induced by various stimuli, such as glucose, KCl, and forskolin, because of absent K_ATP_ channel activity [148]. Heterozygous mutations in *GATA6* [149] and its sister gene *GATA4* [150] can cause neonatal diabetes through pancreatic agenesis. These transcription factors were shown to be essential for pancreatic development while using patient’s iPSCs and iPSCs that are corrected by CRISPR/Cas9 [151,152]. Dominant insulin gene mutations are a common cause of neonatal diabetes [153,154]. These mutations induce insulin protein misfolding, endoplasmic reticulum (ER) stress, and activate the unfolded protein response, which leads to beta cell failure [155,156]. Balboa et al. generated iPSCs from patients with the Akita insulin gene mutation and corrected it using CRISPR/Cas9 [157]. The transplantation of insulin gene mutant beta-like cells into mice resulted in low insulin secretion and the induction of ER stress markers when compared to transplanted corrected beta-like cells. De Franco et al. discovered a neonatal type of diabetes caused by recessive *YIPF5* mutations [158]. They showed increased in vitro sensitivity of patients’ iPSC-derived beta cells to ER stress, which is caused by impaired proinsulin trafficking from the ER to the Golgi, and impaired in vivo function of ESCs edited to express the patients’ mutation.

**Figure 2:**
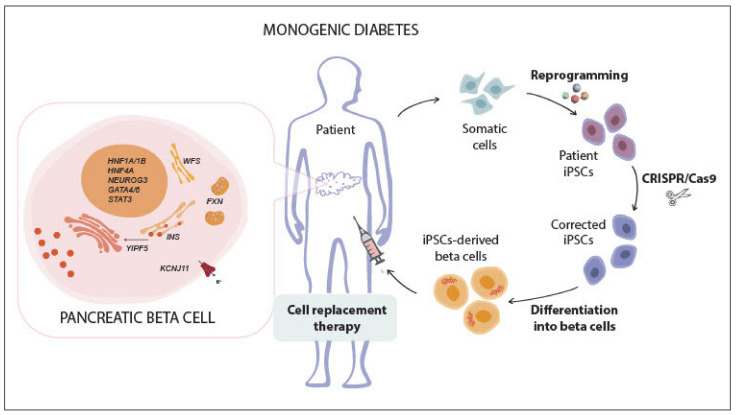
The curative promise of engineered human iPSC-derived beta cells for monogenic diabetes. Monogenic forms of diabetes are caused by mutations in genes critical for beta cell development, function and survival. More specifically, mutations within the *HNF4A*, *HNF1A/1B*, *NEUROG3*, *GATA4/6*, and *STAT3* genes impair beta cell development or pancreatic endocrine development and cause maturity-onset diabetes of the young and neonatal diabetes. Mutations within the *KCNJ11* gene result in dysfunctional K_ATP_-channels, which results in impaired insulin secretion. Furthermore, mutations within the *YIPF5* gene are responsible for impaired proinsulin trafficking from the endoplasmic reticulum to the Golgi apparatus, giving rise to endoplasmic reticulum stress. Interestingly, other rare disorders, such as Friedreich ataxia and the Wolfram syndrome exist that are accompanied by diabetes. Friedreich ataxia is associated with a reduced expression of the *FXN* gene, which causes mitochondrial dysfunction, while Wolfram syndrome is characterized by a dysfunctional *WFS1* gene, which leads to endoplasmic reticulum stress. Since these rare forms of diabetes are usually triggered by a single genetic mutation, it becomes theoretically possible to correct them in patient-derived iPSCs while using CRISPR/Cas9 before their differentiation into healthy beta cells which can be transplanted back into the patient.

Patients with Wolfram syndrome, an autosomal recessive disorder that is caused by *WFS1* mutations, develop childhood diabetes as a result of beta cell ER stress (reviewed in [159,160]). Following CRISPR/Cas9 correction of *WFS1* mutations in patients’ iPSCs, iPSC-beta cells showed dynamic glucose-stimulated insulin secretion and reversed pre-existing streptozotocin-induced diabetes after transplantation into mice [161]. Finally, Friedreich ataxia is an autosomal recessive neurodegenerative disease that is associated with a high risk of developing diabetes. It is caused by intronic GAA trinucleotide repeat expansions in the frataxin-encoding *FXN* gene that reduces frataxin transcription and causes mitochondrial dysfunction [162,163,164,165]. GLP-1 analogs improve mitochondrial function in frataxin-deficient cells and induce frataxin expression in Friedreich ataxia patients’ iPSC-derived neurons and beta-like cells [166]. Taken together, this growing body of evidence shows that the gene editing of iPSCs from patients with monogenic diabetes allows for generating functional beta cells with the potential to provide a personalized cell source for cell replacement therapy (Figure 2).

### 6.2. Where Do the Clinical Trials in T1D Stand?

Hitherto four clinical trials in T1D with stem cell-derived pancreatic cells have been registered, all being initiated by the company ViaCyte. In 2014, the first trial (NCT02239354) was posted on the clinicaltrials.gov platform as a “Prospective, Multicenter, Open-Label, First-in-Human Phase 1/2 Study With Two Cohorts to Evaluate the Safety, Tolerability, and Efficacy of Various Doses of VC-01™ Combination Product in Subjects With Type 1 Diabetes Mellitus”. This VC-01™ combination product consists of a biological product—i.e., pancreatic progenitor cells differentiated from human ESCs (PEC-01)—and a macroencapsulation device. In 2016, a follow-up prospective trial “A One-Year, Multicenter, Observational, Follow-up Safety Study in Subjects Previously Implanted With VC-01™ Combination Product” was posted in order to document the incidence of adverse events that were reported during the study with the VC-01 device. With the estimated final completion dates of NCT02239354 (January 2021) and NCT02939118 (November 2021) approaching, some preliminary results of 19 subjects that have been implanted and evaluated have been reported [14]. These results indicate that the approach is safe and well tolerated with relatively minor adverse events, but, while protection from immune rejection was reported, inconsistent long term graft survival was observed, which is attributed to a foreign body reaction to the device component for which further optimization was due. In 2017, the company launched two more trials. First, NCT03162926 “A Safety and Tolerability Study of VC-02™ Combination Product in Subjects With Type 1 Diabetes Mellitus”, in which their combination product VC-02™ is tested of which the device component is changed from a semi-permeable encapsulation device—that prevents immune cells and blood vessels to enter the biological compartment—to an encapsulation device that does allow for the entry of immune cells and blood vessels (final completion in February 2018). Second, NCT03163511 “A Safety, Tolerability, and Efficacy Study of VC-02™ Combination Product in Subjects With Type 1 Diabetes Mellitus and Hypoglycemia Unawareness”, in which the same combination product is investigated, specifically in patients with hypoglycemia unawareness or significant glycemic lability (estimated completion in March 2023).

Although these trials pave the way for a future functional cure for diabetes, it remains to be evaluated whether the use of pancreatic progenitor cells is the best path if our goal is the full replacement of the functional beta cell mass in patients with diabetes. In order to obtain a true cure and to eliminate the daily burden of diabetes care, the grafted cells will have to reach—at least upon engraftment—full functional maturity in addition to being sufficiently numerous to be able to tightly regulate glucose homeostasis.

## 7. Perspectives

Although current human pluripotent stem cell differentiation protocols succeed in generating beta-like cells that share many phenotypical and functional properties with bona fide beta cells, reaching full functional maturation remains challenging, as: (i) adult beta cells are heterogenous and (ii) current differentiation protocols are based on simplified mimicry of the dynamic and complex interactions of beta cells with other (non)endocrine cells in their native microenvironment. This intercellular communication is likely key to reach, or at least maintain, full beta cell maturity in all of its complexity and heterogeneity.

## Figures and Tables

**Table 1:** Overview of protocols for the generation of stem cell-derived beta cells.

Report	Approach	Outcome to Beta-Cell Function	Reference
Pagliuca et al., 2014	Protocol for generation of beta-like cells by modifying and combining three previous protocols [70,71,72]	Expression of key markers of mature pancreatic beta-cells, glucose-induced Ca^2+^ influx, insulin secretion in response to multiple sequential glucose challenges	[52]
Rezania et al., 2014	7-stage protocol for generation of beta-like cells based on previous own protocol [70]	Expression of key markers of mature pancreatic beta-cells, insulin secretion in response to high glucose	[53]
Russ et al., 2015	Protocol for generation of beta-like cells based on two previous protocols [70] by culture without additional growth factors after endocrine progenitor stage	Expression of key markers of mature pancreatic beta-cells, insulin secretion in response to high glucose	[51]
Millman et al., 2016	Addition of ROCK inhibitor and Activin A based on [52] at pancreatic progenitor stage	Similar to their previous studies [52], beneficial effect on insulin expression and secretion	[56]
Zhu et al., 2016	Addition of vitamin C and BayK-8644 at final stage	Increased insulin expression and secretion	[55]
Ghazizadeh et al., 2017	ROCKII inhibition at pancreatic progenitor stage	Generation and maturation of glucose-responsive cells	[54]
Nair et al., 2019	Reaggregation/clustering after FACS at final stage	Robust dynamic insulin secretion, metabolic maturation by driving mitochondrial oxidative respiration	[57]
Velazco-Cruz et al., 2019	Allowing TGF-β signaling during the final stage and reaggregation/clustering	Pure populations of beta-like cells that secrete high levels of insulin and express key beta cell markers	[58]
Yoshihara et al., 2020	Allowing WNT4 signaling during the final stage	Metabolic maturation with robust insulin secretion and high mitochondrial oxidative respiration	[63]
Li et al., 2020	Combination of three previous protocols [52], and reaggregation/clustering after negative sorting by CD9	High glucose-responsiveness and insulin production	[65]

ROCK: Rho-associated protein kinase, TGF-β: transforming growth factor β, FACS: fluorescence-activated cell sorting, WNT4: wingless-type murine-mammary-tumour virus integration site family member 4, CD9: cluster of differentiation 9.

**Table 2:** Approaches to prevent teratoma formation of stem cell-derived grafts.

Approach	Target	Intervention	Reference
Optimization of differentiation	Oxygen supply	7-stage protocol including culture at air-liquid interface	[53]
	Signaling pathways	Sequential modulation of signaling pathways in a 3D cell culture system	[52]
		Removal of BMP inhibitors in combination with retinoic acid and EGF/KGF addition	[51]
		Modulation of TGF-β signaling	[58]
	Cell clustering	Isolation and reaggregation of immature beta-like cells to form islet-sized beta cell-enriched clusters	[57]
Elimination of remaining undifferentiated cells	Chemical methods	Addition of PluriSln1	[96]
	Immunological methods	Antibodies against SSEA-5 glycan	[97]
		Antibodies against beta cell surface marker CD49a followed by MACS	[66]
		Removal of Claudin-6-positive cells	[99]
		Separation of SSEA-4 and TRA-1-60 undifferentiated cells by MACS and FACS	[98]
	Genetic methods	Double suicide cassette: HSV-TK and NTR	[100]
		Inducible Caspase-9 suicide gene	[101]

BMP: bone morphogenetic proteins, EGF: epidermal growth factor, FACS: fluorescence-activated cell sorting, HSV-TK: herpes simplex virus thymidine kinase, KGF: keratinocyte growth factor, MACS: magnetic-activated cell sorting, NTR: nitroreductase, PluriSln1: pluripotent cell-specific inhibitors, SSEA: stage-specific embryonic antigen, TGF-β: transforming growth factor β.

**Table 3:** Approaches to prevent immune rejection of stem cell-derived grafts.

Point of Action	Approach	Intervention	Species (Graft to Host)	Outcome	Reference
Graft	Hypo-immunity	Deletion of β2M	B2M knock-out ESCs transplanted in NK-cell depleted mice	(+) Prevents immune rejection, confers resistance to T-cell mediated killing	[124]
				(−) Loss of NK-tolerance	
		Deletion of β2M and CIITA	HLA-1/2 knock-out iPSCs *in vitro*	(+) Eliminates immunogenicity, universal donor cell therapy potential	[122]
				(−) Loss of NK-tolerance	
		Deletion of HLA-A/-B/-C and CIITA + PD-L1, HLA-G and CD47 upregulation	Engineered human stem cells transplanted in mice	(+) Less immune activation, decreases T–& NK-cell mediated killing and macrophage engulfment	[123]
				(−) Complex genetic engineering, risk of off-target events	
	Immune-checkpoint modulation	SA-PD-L1 engineered islet grafts	SA-PDL1 mouse islets transplanted in mice	(+) Sustains graft survival, prevents allograft immune rejection, confers localized immunomodulation	[125]
				(−) Short rapamycin treatment for long-term graft survival	
		CTLA-4Ig and PD-L1 knock-in	Knock-in human ESCs transplanted in humanized mice	(+) Protects from allogeneic immune response	[126]
				(−) Escape from immune surveillance (by grafted tumorigenic cells and viral infections)	
	Graft supplementation	PD-L1 microgels	Allogeneic mouse islets co-transplanted with PD-L1 microgels in mice	(+) Prevents graft rejection, promotes tolerance, confers local immuno-protective response, off-the-shelf immunomodulation strategy	[127]
				(−) Some recipients only partial benefit, short low-dose rapamycin treatment to fully prevent rejection	
		MSCs	Allograft islets in combination with MSCs transplanted in mice, rats and non-human primates	(+) Downregulates pro-inflammatory cytokines, prevents allograft immune rejection, promotes graft survival	[114,115,116,117]
				(−) Logistical and regulatory concerns–clinical transplantation via hepatic portal vein limits co-engraftment	
		MSC-derived cell-free cocktail	Pre-treated mouse islets transplanted in diabetic mice	(+) Protects from cytokine-mediated cell death in vitro, improves functional graft survival in vivo	[120]
Host	Treg therapy	*Ex vivo* expanded polyclonal or antigen-specific Tregs	Tregs injected systemically in mice and T1D patients	(+) Prolongs islet allograft survival, inhibits alloimmune response in mice, clinical trials demonstrated feasibility and safety in human	[106,107,108,128]
				(−) Poor engraftment, Treg manufacturing is challenging	
	Production of anti-inflammatory cytokines	IL-10 secretion by designer beta cells	Glucose-dependent secretion of IL-10 by murine beta cells	(+) Protects from pro-inflammatory cytokine-induced cell death, minimal systemic effects on host immune system, efficient engineering	[111]
	Immune-checkpoint modulation	Administration of CTLA-4Ig and anti CD40L mAbs	ESC-PE transplanted in (humanized) mice	(+) Prevents immune rejection, induces immune tolerance, prolongs graft survival	[129]
				(−) Testing only possible in immunocompromised animals (limited to rodents)	

(+): advantages, (−) disadvantages. β2M: beta 2 microglobulin, CD: cluster of Differentiation, CIITA: class II Major Histocompatibility Complex Transactivator, CTLA: cytotoxic T Lymphocyte-Associated antigen, hESCs: human embryonic stem cells, hESC-PE: human embryonic stem-cell-derived pancreatic endoderm, Hi-PSCs: human induced pluripotent stem cells, hPSCs: human pluripotent stem cells, HLA: human leukocyte antigens, Ig: immunoglobulin, IL: interleukin, mAbs: monoclonal antibodies, MSCs: mesenchymal stem cells, NK: natural killer, PD-L: programmed death-ligand, SA: streptavidin, Treg: regulatory T-cell.

## Data Availability

Not applicable.
